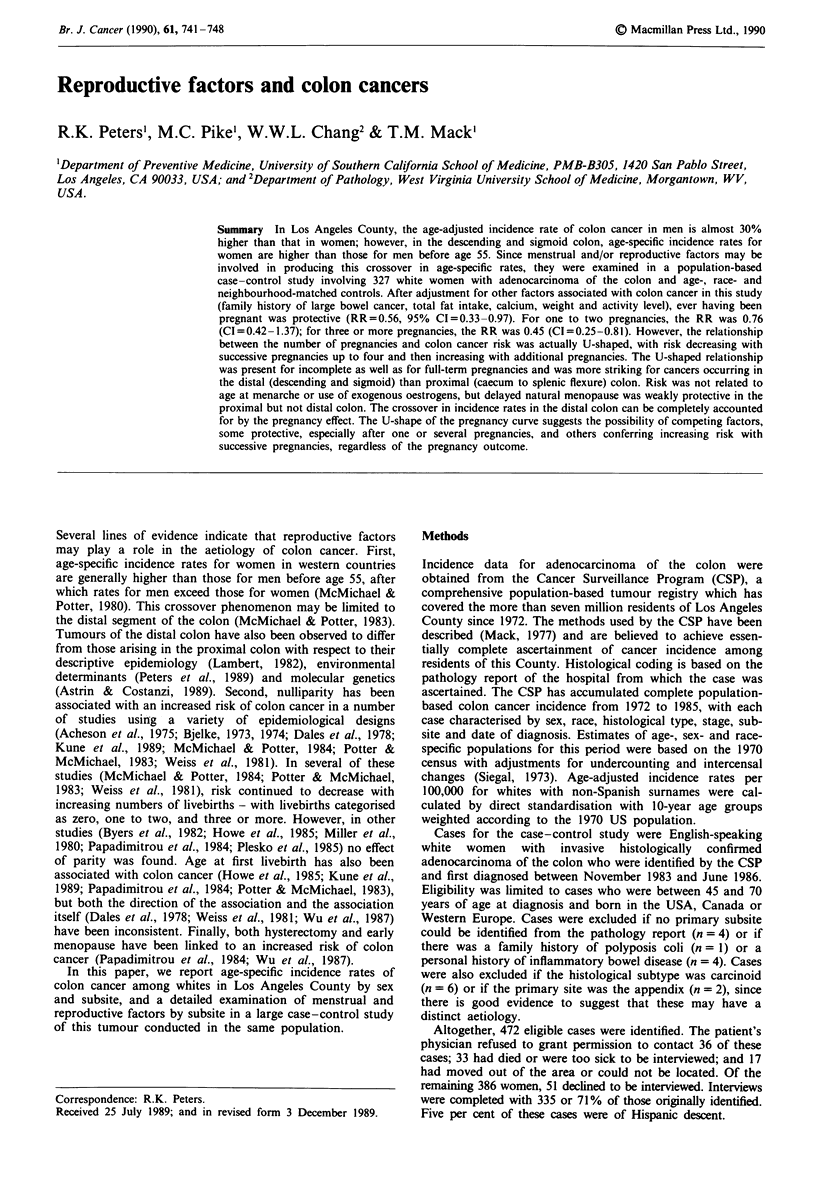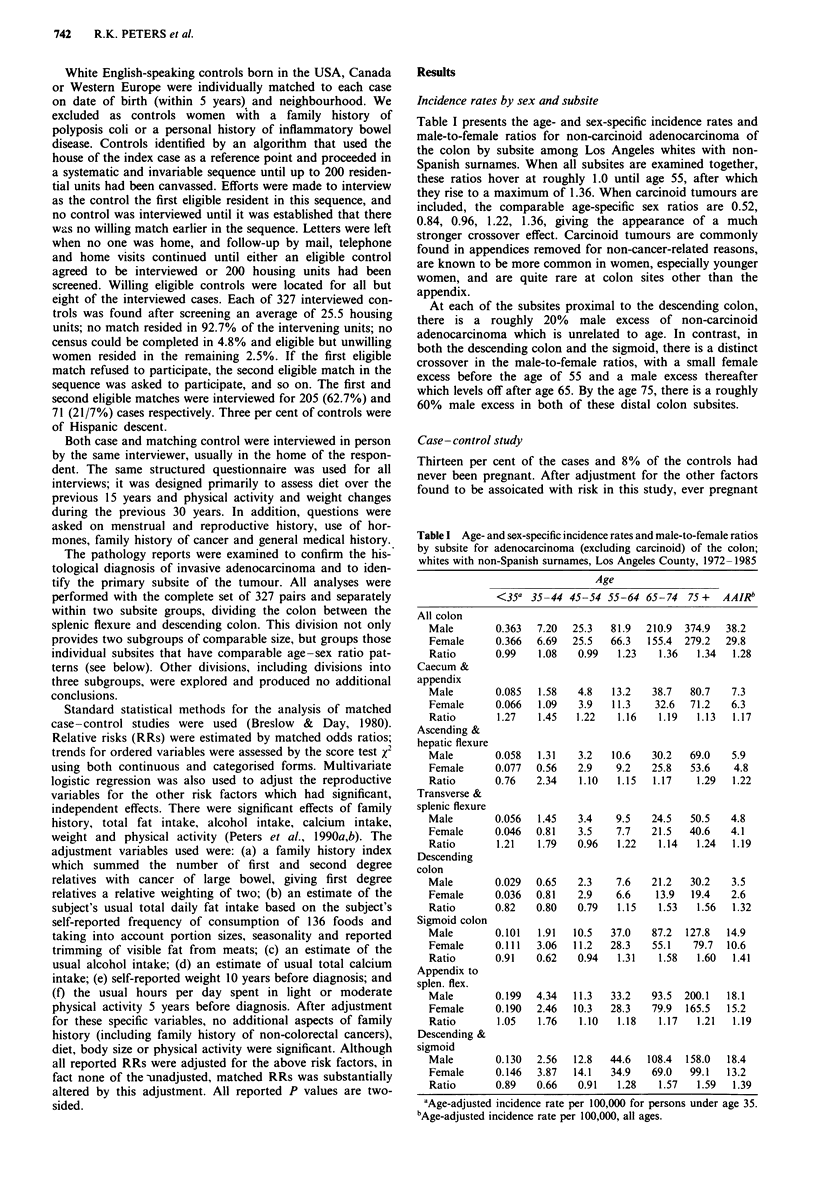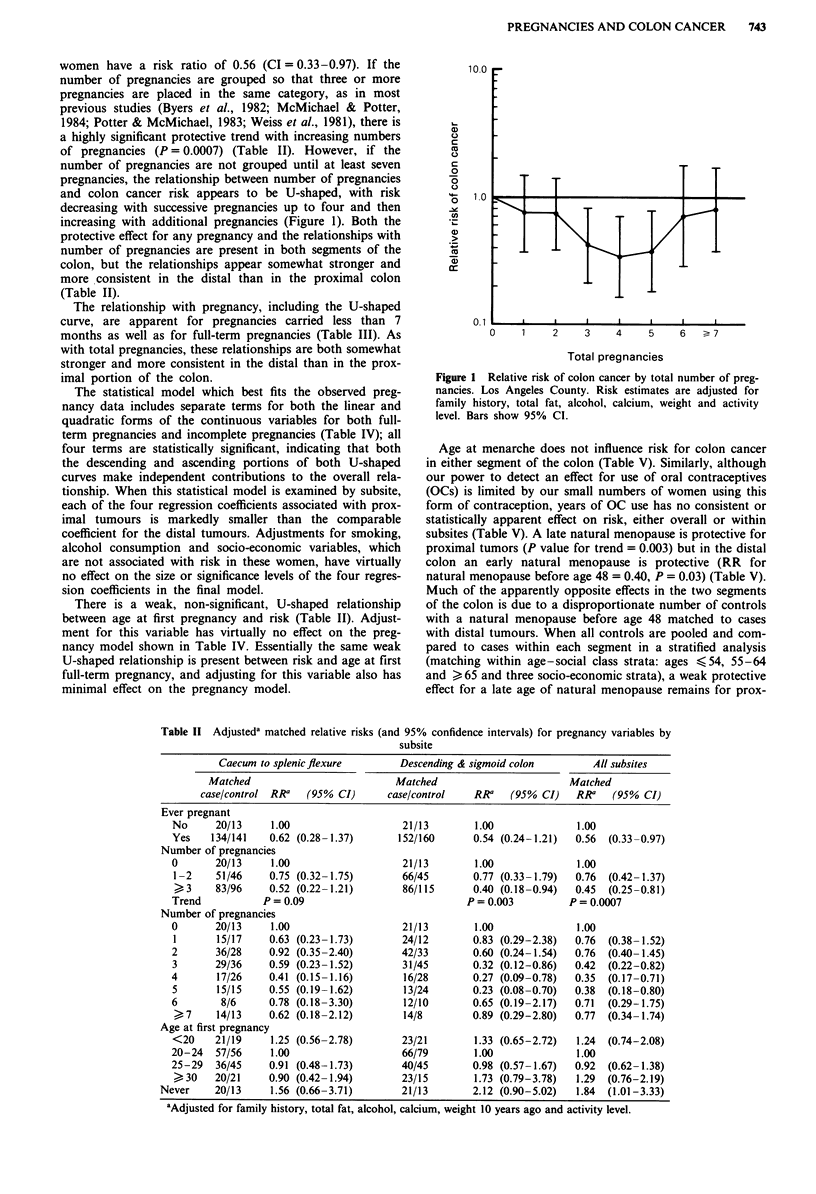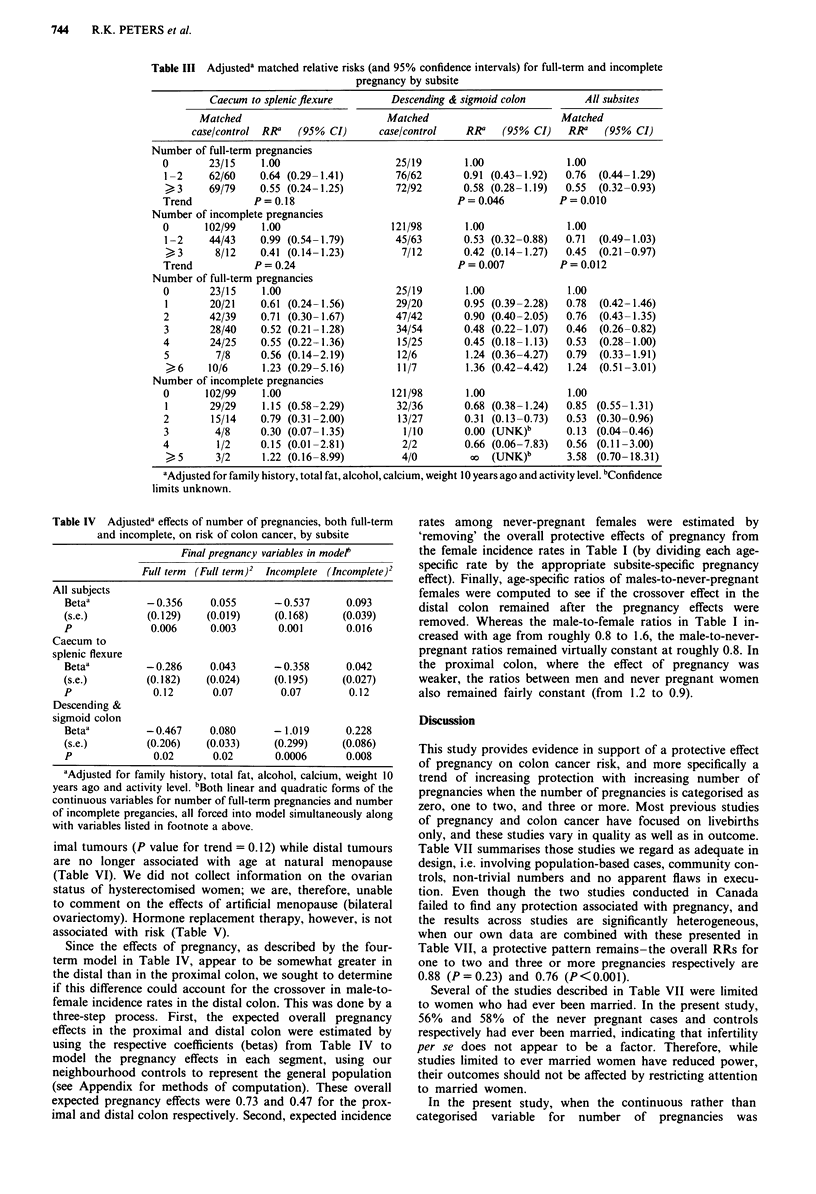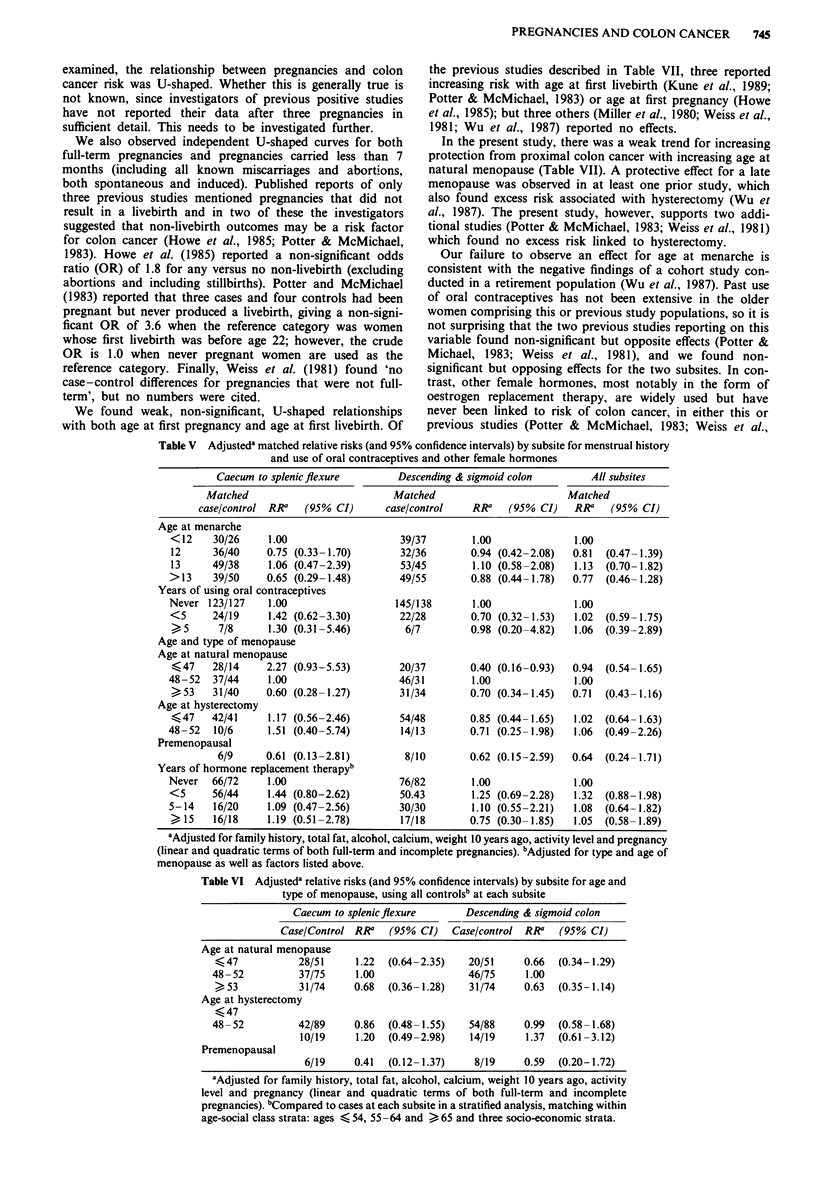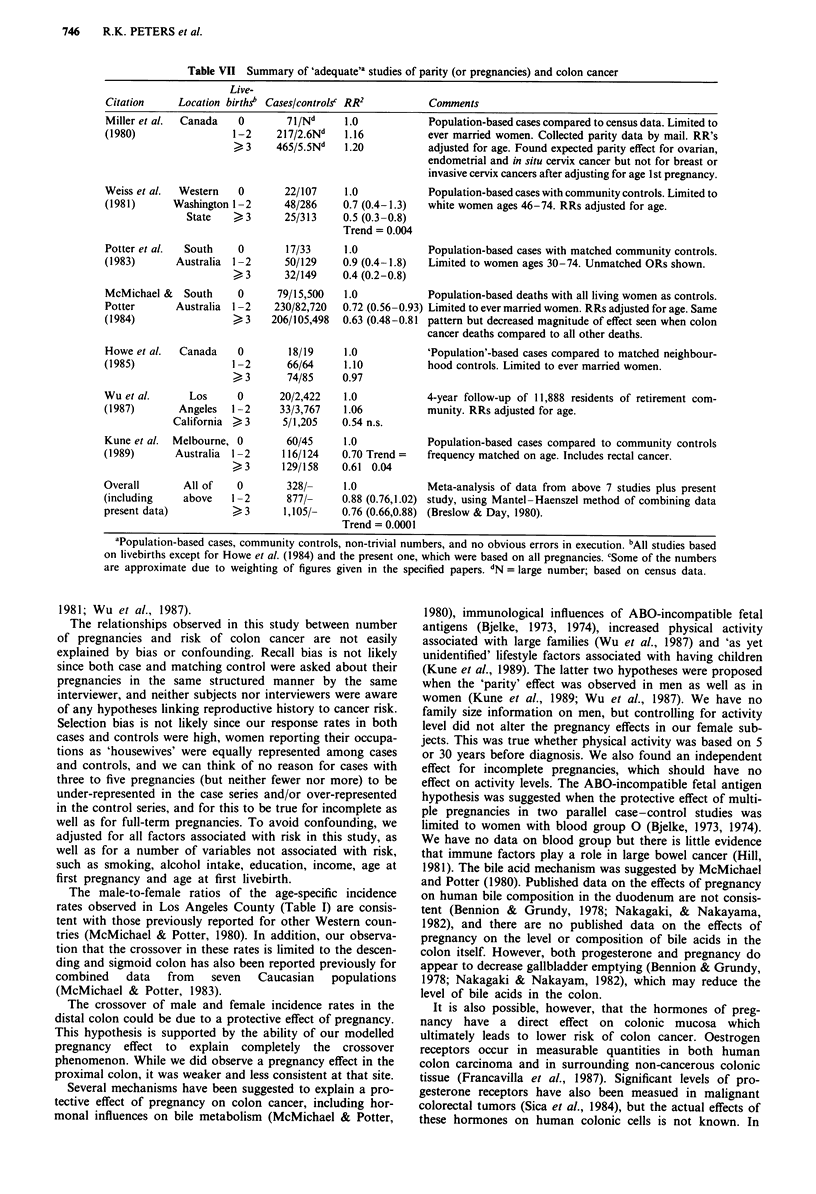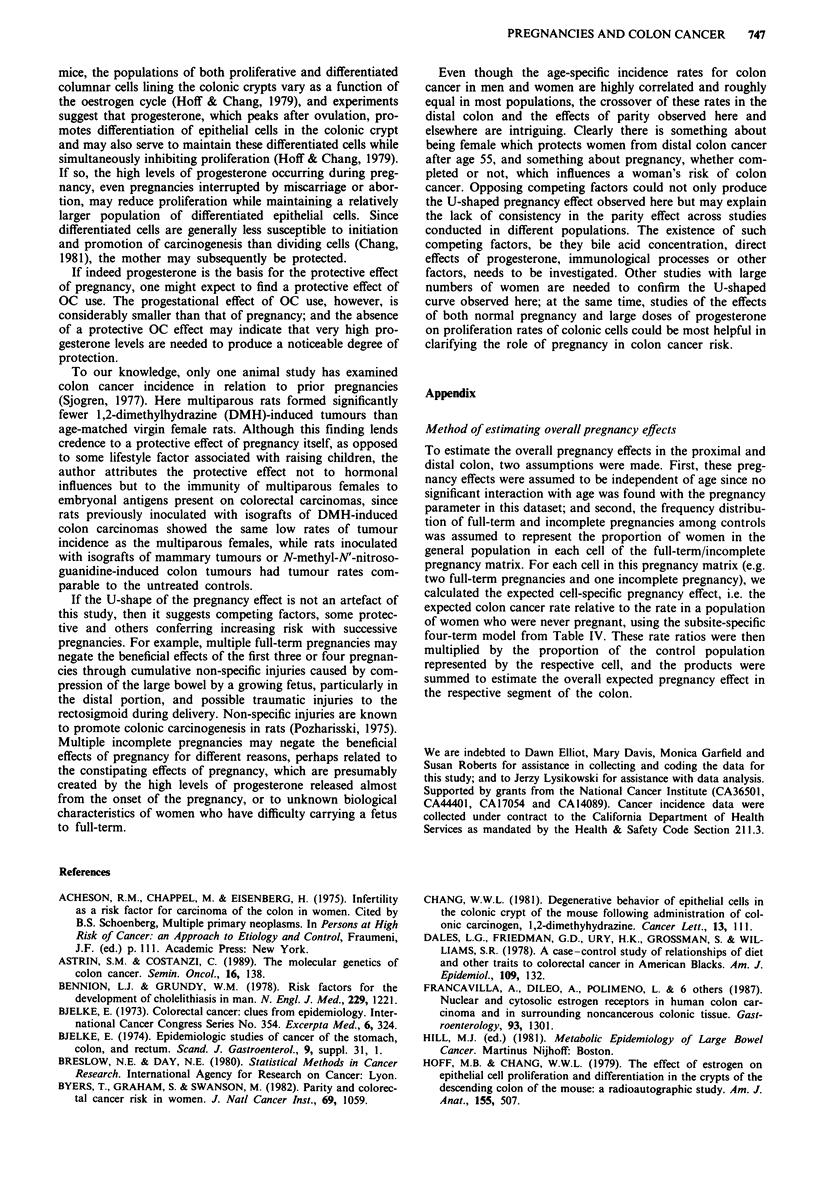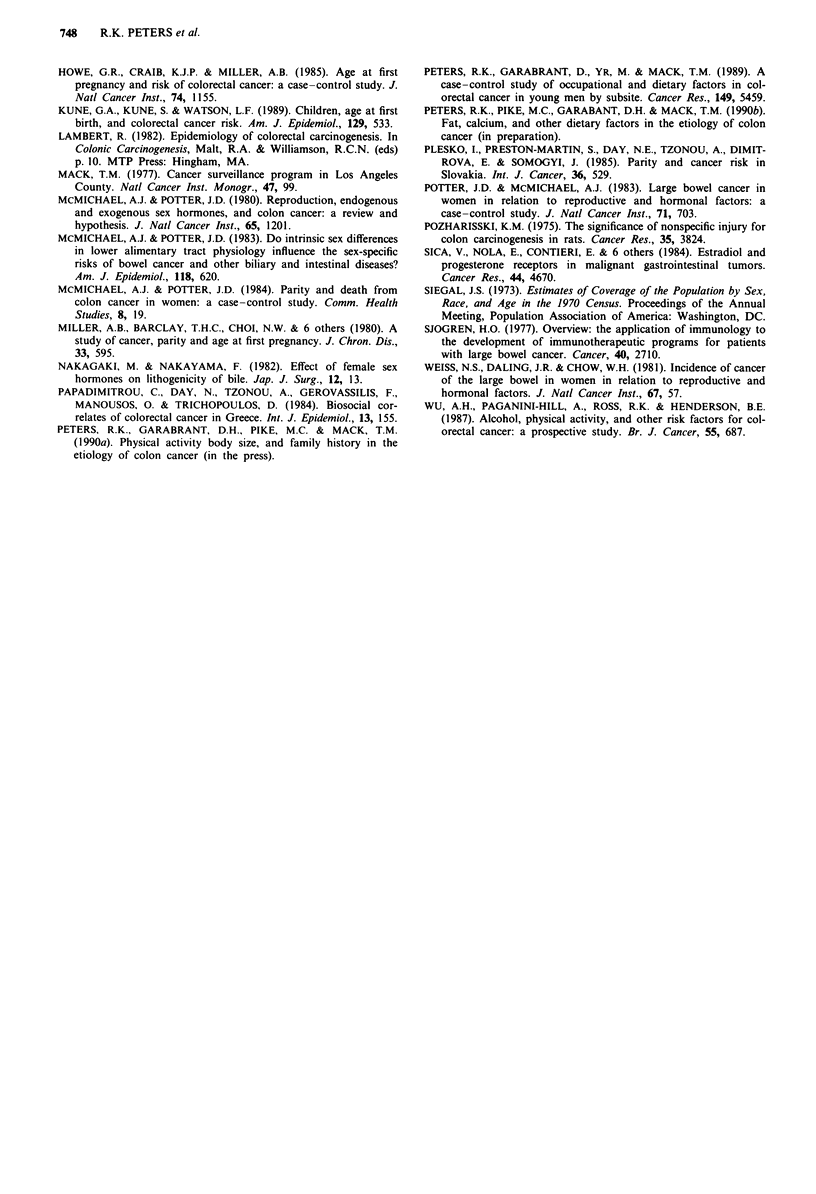# Reproductive factors and colon cancers.

**DOI:** 10.1038/bjc.1990.166

**Published:** 1990-05

**Authors:** R. K. Peters, M. C. Pike, W. W. Chang, T. M. Mack

**Affiliations:** Department of Preventive Medicine, University of Southern California School of Medicine, Los Angeles 90033.

## Abstract

In Los Angeles County, the age-adjusted incidence rate of colon cancer in men is almost 30% higher than that in women; however, in the descending and sigmoid colon, age-specific incidence rates for women are higher than those for men before age 55. Since menstrual and/or reproductive factors may be involved in producing this crossover in age-specific rates, they were examined in a population-based case-control study involving 327 white women with adenocarcinoma of the colon and age-, race- and neighbourhood-matched controls. After adjustment for other factors associated with colon cancer in this study (family history of large bowel cancer, total fat intake, calcium, weight and activity level), ever having been pregnant was protective (RR = 0.56, 95% CI = 0.33-0.97). For one to two pregnancies, the RR was 0.76 (CI = 0.42-1.37); for three or more pregnancies, the RR was 0.45 (CI = 0.25-0.81). However, the relationship between the number of pregnancies and colon cancer risk was actually U-shaped, with risk decreasing with successive pregnancies up to four and then increasing with additional pregnancies. The U-shaped relationship was present for incomplete as well as for full-term pregnancies and was more striking for cancers occurring in the distal (descending and sigmoid) than proximal (caecum to splenic flexure) colon. Risk was not related to age at menarche or use of exogenous oestrogens, but delayed natural menopause was weakly protective in the proximal but not distal colon. The crossover in incidence rates in the distal colon can be completely accounted for by the pregnancy effect. The U-shape of the pregnancy curve suggests the possibility of competing factors, some protective, especially after one or several pregnancies, and others conferring increasing risk with successive pregnancies, regardless of the pregnancy outcome.


Br. J. Cancer (1990), 61, 741-748 © Macmillan Press Ltd., 1990

### Reproductive factors and colon cancers

R.K. Peters[1], M.C. Pike[1], W.W.L. Chang[2] & T.M. Mack[1]

[1]*Department of Preventive Medicine, University of Southern California School of Medicine, PMB-B305, 1420 San Pablo Street, Los Angeles, CA 90033, USA; and* [2]*Department of Pathology, West Virginia University School of Medicine, Morgantown, WV, USA.*

**Summary** In Los Angeles County, the age-adjusted incidence rate of colon cancer in men is almost 30% higher than that in women; however, in the descending and sigmoid colon, age-specific incidence rates for women are higher than those for men before age 55. Since menstrual and/or reproductive factors may be involved in producing this crossover in age-specific rates, they were examined in a population-based case-control study involving 327 white women with adenocarcinoma of the colon and age-, race- and neighbourhood-matched controls. After adjustment for other factors associated with colon cancer in this study (family history of large bowel cancer, total fat intake, calcium, weight and activity level), ever having been pregnant was protective (RR = 0.56, 95% CI = 0.33-0.97). For one to two pregnancies, the RR was 0.76 (CI = 0.42-1.37); for three or more pregnancies, the RR was 0.45 (CI = 0.25-0.81). However, the relationship between the number of pregnancies and colon cancer risk was actually U-shaped, with risk decreasing with successive pregnancies up to four and then increasing with additional pregnancies. The U-shaped relationship was present for incomplete as well as for full-term pregnancies and was more striking for cancers occurring in the distal (descending and sigmoid) than proximal (caecum to splenic flexure) colon. Risk was not related to age at menarche or use of exogenous oestrogens, but delayed natural menopause was weakly protective in the proximal but not distal colon. The crossover in incidence rates in the distal colon can be completely accounted for by the pregnancy effect. The U-shape of the pregnancy curve suggests the possibility of competing factors, some protective, especially after one or several pregnancies, and others conferring increasing risk with successive pregnancies, regardless of the pregnancy outcome.

Several lines of evidence indicate that reproductive factors may play a role in the aetiology of colon cancer. First, age-specific incidence rates for women in western countries are generally higher than those for men before age 55, after which rates for men exceed those for women (McMichael & Potter, 1980). This crossover phenomenon may be limited to the distal segment of the colon (McMichael & Potter, 1983). Tumours of the distal colon have also been observed to differ from those arising in the proximal colon with respect to their descriptive epidemiology (Lambert, 1982), environmental determinants (Peters *et al.*, 1989) and molecular genetics (Astrin & Costanzi, 1989). Second, nulliparity has been associated with an increased risk of colon cancer in a number of studies using a variety of epidemiological designs (Acheson *et al.*, 1975; Bjelke, 1973, 1974; Dales *et al.*, 1978; Kune *et al.*, 1989; McMichael & Potter, 1984; Potter & McMichael, 1983; Weiss *et al.*, 1981). In several of these studies (McMichael & Potter, 1984; Potter & McMichael, 1983; Weiss *et al.*, 1981), risk continued to decrease with increasing numbers of livebirths - with livebirths categorised as zero, one to two, and three or more. However, in other studies (Byers *et al.*, 1982; Howe *et al.*, 1985; Miller *et al.*, 1980; Papadimitrou *et al.*, 1984; Plesko *et al.*, 1985) no effect of parity was found. Age at first livebirth has also been associated with colon cancer (Howe *et al.*, 1985; Kune *et al.*, 1989; Papadimitrou *et al.*, 1984; Potter & McMichael, 1983), but both the direction of the association and the association itself (Dales *et al.*, 1978; Weiss *et al.*, 1981; Wu *et al.*, 1987) have been inconsistent. Finally, both hysterectomy and early menopause have been linked to an increased risk of colon cancer (Papadimitrou *et al.*, 1984; Wu *et al.*, 1987).

In this paper, we report age-specific incidence rates of colon cancer among whites in Los Angeles County by sex and subsite, and a detailed examination of menstrual and reproductive factors by subsite in a large case-control study of this tumour conducted in the same population.

Correspondence: R.K. Peters.
Received 25 July 1989; and in revised form 3 December 1989.

### Methods

Incidence data for adenocarcinoma of the colon were obtained from the Cancer Surveillance Program (CSP), a comprehensive population-based tumour registry which has covered the more than seven million residents of Los Angeles County since 1972. The methods used by the CSP have been described (Mack, 1977) and are believed to achieve essentially complete ascertainment of cancer incidence among residents of this County. Histological coding is based on the pathology report of the hospital from which the case was ascertained. The CSP has accumulated complete population-based colon cancer incidence from 1972 to 1985, with each case characterised by sex, race, histological type, stage, subsite and date of diagnosis. Estimates of age-, sex- and race-specific populations for this period were based on the 1970 census with adjustments for undercounting and intercensal changes (Siegal, 1973). Age-adjusted incidence rates per 100,000 for whites with non-Spanish surnames were calculated by direct standardisation with 10-year age groups weighted according to the 1970 US population.

Cases for the case-control study were English-speaking white women with invasive histologically confirmed adenocarcinoma of the colon who were identified by the CSP and first diagnosed between November 1983 and June 1986. Eligibility was limited to cases who were between 45 and 70 years of age at diagnosis and born in the USA, Canada or Western Europe. Cases were excluded if no primary subsite could be identified from the pathology report ($n = 4$) or if there was a family history of polyposis coli ($n = 1$) or a personal history of inflammatory bowel disease ($n = 4$). Cases were also excluded if the histological subtype was carcinoid ($n = 6$) or if the primary site was the appendix ($n = 2$), since there is good evidence to suggest that these may have a distinct aetiology.

Altogether, 472 eligible cases were identified. The patient's physician refused to grant permission to contact 36 of these cases; 33 had died or were too sick to be interviewed; and 17 had moved out of the area or could not be located. Of the remaining 386 women, 51 declined to be interviewed. Interviews were completed with 335 or 71% of those originally identified. Five per cent of these cases were of Hispanic descent.

White English-speaking controls born in the USA, Canada or Western Europe were individually matched to each case on date of birth (within 5 years) and neighbourhood. We excluded as controls women with a family history of polyposis coli or a personal history of inflammatory bowel disease. Controls identified by an algorithm that used the house of the index case as a reference point and proceeded in a systematic and invariable sequence until up to 200 residential units had been canvassed. Efforts were made to interview as the control the first eligible resident in this sequence, and no control was interviewed until it was established that there was no willing match earlier in the sequence. Letters were left when no one was home, and follow-up by mail, telephone and home visits continued until either an eligible control agreed to be interviewed or 200 housing units had been screened. Willing eligible controls were located for all but eight of the interviewed cases. Each of 327 interviewed controls was found after screening an average of 25.5 housing units; no match resided in 92.7% of the intervening units; no census could be completed in 4.8% and eligible but unwilling women resided in the remaining 2.5%. If the first eligible match refused to participate, the second eligible match in the sequence was asked to participate, and so on. The first and second eligible matches were interviewed for 205 (62.7%) and 71 (21/7%) cases respectively. Three per cent of controls were of Hispanic descent.

Both case and matching control were interviewed in person by the same interviewer, usually in the home of the respondent. The same structured questionnaire was used for all interviews; it was designed primarily to assess diet over the previous 15 years and physical activity and weight changes during the previous 30 years. In addition, questions were asked on menstrual and reproductive history, use of hormones, family history of cancer and general medical history.

The pathology reports were examined to confirm the histological diagnosis of invasive adenocarcinoma and to identify the primary subsite of the tumour. All analyses were performed with the complete set of 327 pairs and separately within two subsite groups, dividing the colon between the splenic flexure and descending colon. This division not only provides two subgroups of comparable size, but groups those individual subsites that have comparable age–sex ratio patterns (see below). Other divisions, including divisions into three subgroups, were explored and produced no additional conclusions.

Standard statistical methods for the analysis of matched case–control studies were used (Breslow & Day, 1980). Relative risks (RRs) were estimated by matched odds ratios; trends for ordered variables were assessed by the score test $\chi^2$ using both continuous and categorised forms. Multivariate logistic regression was also used to adjust the reproductive variables for the other risk factors which had significant, independent effects. There were significant effects of family history, total fat intake, alcohol intake, calcium intake, weight and physical activity (Peters *et al.*, 1990*a*,*b*). The adjustment variables used were: (a) a family history index which summed the number of first and second degree relatives with cancer of large bowel, giving first degree relatives a relative weighting of two; (b) an estimate of the subject's usual total daily fat intake based on the subject's self-reported frequency of consumption of 136 foods and taking into account portion sizes, seasonality and reported trimming of visible fat from meats; (c) an estimate of the usual alcohol intake; (d) an estimate of usual total calcium intake; (e) self-reported weight 10 years before diagnosis; and (f) the usual hours per day spent in light or moderate physical activity 5 years before diagnosis. After adjustment for these specific variables, no additional aspects of family history (including family history of non-colorectal cancers), diet, body size or physical activity were significant. Although all reported RRs were adjusted for the above risk factors, in fact none of the unadjusted, matched RRs was substantially altered by this adjustment. All reported *P* values are two-sided.

### Results

### *Incidence rates by sex and subsite*

Table I presents the age- and sex-specific incidence rates and male-to-female ratios for non-carcinoid adenocarcinoma of the colon by subsite among Los Angeles whites with non-Spanish surnames. When all subsites are examined together, these ratios hover at roughly 1.0 until age 55, after which they rise to a maximum of 1.36. When carcinoid tumours are included, the comparable age-specific sex ratios are 0.52, 0.84, 0.96, 1.22, 1.36, giving the appearance of a much stronger crossover effect. Carcinoid tumours are commonly found in appendices removed for non-cancer-related reasons, are known to be more common in women, especially younger women, and are quite rare at colon sites other than the appendix.

At each of the subsites proximal to the descending colon, there is a roughly 20% male excess of non-carcinoid adenocarcinoma which is unrelated to age. In contrast, in both the descending colon and the sigmoid, there is a distinct crossover in the male-to-female ratios, with a small female excess before the age of 55 and a male excess thereafter which levels off after age 65. By the age 75, there is a roughly 60% male excess in both of these distal colon subsites.

### *Case–control study*

Thirteen per cent of the cases and 8% of the controls had never been pregnant. After adjustment for the other factors found to be assoicated with risk in this study, ever pregnant

**Table I** Age- and sex-specific incidence rates and male-to-female ratios by subsite for adenocarcinoma (excluding carcinoid) of the colon; whites with non-Spanish surnames, Los Angeles County, 1972–1985

| | *Age* | | | | | | |
|---|---|---|---|---|---|---|---|
| | *<35*[a] | *35–44* | *45–54* | *55–64* | *65–74* | *75 +* | *AAIR*[b] |
| All colon | | | | | | | |
| Male | 0.363 | 7.20 | 25.3 | 81.9 | 210.9 | 374.9 | 38.2 |
| Female | 0.366 | 6.69 | 25.5 | 66.3 | 155.4 | 279.2 | 29.8 |
| Ratio | 0.99 | 1.08 | 0.99 | 1.23 | 1.36 | 1.34 | 1.28 |
| Caecum & appendix | | | | | | | |
| Male | 0.085 | 1.58 | 4.8 | 13.2 | 38.7 | 80.7 | 7.3 |
| Female | 0.066 | 1.09 | 3.9 | 11.3 | 32.6 | 71.2 | 6.3 |
| Ratio | 1.27 | 1.45 | 1.22 | 1.16 | 1.19 | 1.13 | 1.17 |
| Ascending & hepatic flexure | | | | | | | |
| Male | 0.058 | 1.31 | 3.2 | 10.6 | 30.2 | 69.0 | 5.9 |
| Female | 0.077 | 0.56 | 2.9 | 9.2 | 25.8 | 53.6 | 4.8 |
| Ratio | 0.76 | 2.34 | 1.10 | 1.15 | 1.17 | 1.29 | 1.22 |
| Transverse & splenic flexure | | | | | | | |
| Male | 0.056 | 1.45 | 3.4 | 9.5 | 24.5 | 50.5 | 4.8 |
| Female | 0.046 | 0.81 | 3.5 | 7.7 | 21.5 | 40.6 | 4.1 |
| Ratio | 1.21 | 1.79 | 0.96 | 1.22 | 1.14 | 1.24 | 1.19 |
| Descending colon | | | | | | | |
| Male | 0.029 | 0.65 | 2.3 | 7.6 | 21.2 | 30.2 | 3.5 |
| Female | 0.036 | 0.81 | 2.9 | 6.6 | 13.9 | 19.4 | 2.6 |
| Ratio | 0.82 | 0.80 | 0.79 | 1.15 | 1.53 | 1.56 | 1.32 |
| Sigmoid colon | | | | | | | |
| Male | 0.101 | 1.91 | 10.5 | 37.0 | 87.2 | 127.8 | 14.9 |
| Female | 0.111 | 3.06 | 11.2 | 28.3 | 55.1 | 79.7 | 10.6 |
| Ratio | 0.91 | 0.62 | 0.94 | 1.31 | 1.58 | 1.60 | 1.41 |
| Appendix to splen. flex. | | | | | | | |
| Male | 0.199 | 4.34 | 11.3 | 33.2 | 93.5 | 200.1 | 18.1 |
| Female | 0.190 | 2.46 | 10.3 | 28.3 | 79.9 | 165.5 | 15.2 |
| Ratio | 1.05 | 1.76 | 1.10 | 1.18 | 1.17 | 1.21 | 1.19 |
| Descending & sigmoid | | | | | | | |
| Male | 0.130 | 2.56 | 12.8 | 44.6 | 108.4 | 158.0 | 18.4 |
| Female | 0.146 | 3.87 | 14.1 | 34.9 | 69.0 | 99.1 | 13.2 |
| Ratio | 0.89 | 0.66 | 0.91 | 1.28 | 1.57 | 1.59 | 1.39 |

[a]Age-adjusted incidence rate per 100,000 for persons under age 35.
[b]Age-adjusted incidence rate per 100,000, all ages.

women have a risk ratio of 0.56 (CI = 0.33–0.97). If the number of pregnancies are grouped so that three or more pregnancies are placed in the same category, as in most previous studies (Byers *et al.*, 1982; McMichael & Potter, 1984; Potter & McMichael, 1983; Weiss *et al.*, 1981), there is a highly significant protective trend with increasing numbers of pregnancies ($P = 0.0007$) (Table II). However, if the number of pregnancies are not grouped until at least seven pregnancies, the relationship between number of pregnancies and colon cancer risk appears to be U-shaped, with risk decreasing with successive pregnancies up to four and then increasing with additional pregnancies (Figure 1). Both the protective effect for any pregnancy and the relationships with number of pregnancies are present in both segments of the colon, but the relationships appear somewhat stronger and more consistent in the distal than in the proximal colon (Table II).

The relationship with pregnancy, including the U-shaped curve, are apparent for pregnancies carried less than 7 months as well as for full-term pregnancies (Table III). As with total pregnancies, these relationships are both somewhat stronger and more consistent in the distal than in the proximal portion of the colon.

The statistical model which best fits the observed pregnancy data includes separate terms for both the linear and quadratic forms of the continuous variables for both full-term pregnancies and incomplete pregnancies (Table IV); all four terms are statistically significant, indicating that both the descending and ascending portions of both U-shaped curves make independent contributions to the overall relationship. When this statistical model is examined by subsite, each of the four regression coefficients associated with proximal tumours is markedly smaller than the comparable coefficient for the distal tumours. Adjustments for smoking, alcohol consumption and socio-economic variables, which are not associated with risk in these women, have virtually no effect on the size or significance levels of the four regression coefficients in the final model.

There is a weak, non-significant, U-shaped relationship between age at first pregnancy and risk (Table II). Adjustment for this variable has virtually no effect on the pregnancy model shown in Table IV. Essentially the same weak U-shaped relationship is present between risk and age at first full-term pregnancy, and adjusting for this variable also has minimal effect on the pregnancy model.

**Figure 1** Relative risk of colon cancer by total number of pregnancies. Los Angeles County. Risk estimates are adjusted for family history, total fat, alcohol, calcium, weight and activity level. Bars show 95% CI.

Age at menarche does not influence risk for colon cancer in either segment of the colon (Table V). Similarly, although our power to detect an effect for use of oral contraceptives (OCs) is limited by our small numbers of women using this form of contraception, years of OC use has no consistent or statistically apparent effect on risk, either overall or within subsites (Table V). A late natural menopause is protective for proximal tumors ($P$ value for trend = 0.003) but in the distal colon an early natural menopause is protective (RR for natural menopause before age 48 = 0.40, $P = 0.03$) (Table V). Much of the apparently opposite effects in the two segments of the colon is due to a disproportionate number of controls with a natural menopause before age 48 matched to cases with distal tumours. When all controls are pooled and compared to cases within each segment in a stratified analysis (matching within age–social class strata: ages ⩽54, 55–64 and ⩾65 and three socio-economic strata), a weak protective effect for a late age of natural menopause remains for prox-

**Table II** Adjusted[a] matched relative risks (and 95% confidence intervals) for pregnancy variables by subsite

| | *Caecum to splenic flexure* | | | *Descending & sigmoid colon* | | | *All subsites* | |
|---|---|---|---|---|---|---|---|---|
| | *Matched case/control* | *RR*[a] | *(95% CI)* | *Matched case/control* | *RR*[a] | *(95% CI)* | *Matched RR*[a] | *(95% CI)* |
| Ever pregnant | | | | | | | | |
| No | 20/13 | 1.00 | | 21/13 | 1.00 | | 1.00 | |
| Yes | 134/141 | 0.62 | (0.28–1.37) | 152/160 | 0.54 | (0.24–1.21) | 0.56 | (0.33–0.97) |
| Number of pregnancies | | | | | | | | |
| 0 | 20/13 | 1.00 | | 21/13 | 1.00 | | 1.00 | |
| 1–2 | 51/46 | 0.75 | (0.32–1.75) | 66/45 | 0.77 | (0.33–1.79) | 0.76 | (0.42–1.37) |
| ⩾3 | 83/96 | 0.52 | (0.22–1.21) | 86/115 | 0.40 | (0.18–0.94) | 0.45 | (0.25–0.81) |
| Trend | | $P = 0.09$ | | | $P = 0.003$ | | $P = 0.0007$ | |
| Number of pregnancies | | | | | | | | |
| 0 | 20/13 | 1.00 | | 21/13 | 1.00 | | 1.00 | |
| 1 | 15/17 | 0.63 | (0.23–1.73) | 24/12 | 0.83 | (0.29–2.38) | 0.76 | (0.38–1.52) |
| 2 | 36/28 | 0.92 | (0.35–2.40) | 42/33 | 0.60 | (0.24–1.54) | 0.76 | (0.40–1.45) |
| 3 | 29/36 | 0.59 | (0.23–1.52) | 31/45 | 0.32 | (0.12–0.86) | 0.42 | (0.22–0.82) |
| 4 | 17/26 | 0.41 | (0.15–1.16) | 16/28 | 0.27 | (0.09–0.78) | 0.35 | (0.17–0.71) |
| 5 | 15/15 | 0.55 | (0.19–1.62) | 13/24 | 0.23 | (0.08–0.70) | 0.38 | (0.18–0.80) |
| 6 | 8/6 | 0.78 | (0.18–3.30) | 12/10 | 0.65 | (0.19–2.17) | 0.71 | (0.29–1.75) |
| ⩾7 | 14/13 | 0.62 | (0.18–2.12) | 14/8 | 0.89 | (0.29–2.80) | 0.77 | (0.34–1.74) |
| Age at first pregnancy | | | | | | | | |
| <20 | 21/19 | 1.25 | (0.56–2.78) | 23/21 | 1.33 | (0.65–2.72) | 1.24 | (0.74–2.08) |
| 20–24 | 57/56 | 1.00 | | 66/79 | 1.00 | | 1.00 | |
| 25–29 | 36/45 | 0.91 | (0.48–1.73) | 40/45 | 0.98 | (0.57–1.67) | 0.92 | (0.62–1.38) |
| ⩾30 | 20/21 | 0.90 | (0.42–1.94) | 23/15 | 1.73 | (0.79–3.78) | 1.29 | (0.76–2.19) |
| Never | 20/13 | 1.56 | (0.66–3.71) | 21/13 | 2.12 | (0.90–5.02) | 1.84 | (1.01–3.33) |

[a]Adjusted for family history, total fat, alcohol, calcium, weight 10 years ago and activity level.

**Table III** Adjusted[a] matched relative risks (and 95% confidence intervals) for full-term and incomplete pregnancy by subsite

| | *Caecum to splenic flexure* | | | *Descending & sigmoid colon* | | | *All subsites* | |
|---|---|---|---|---|---|---|---|---|
| | *Matched case/control* | *RR*[a] | *(95% CI)* | *Matched case/control* | *RR*[a] | *(95% CI)* | *RR*[a] | *(95% CI)* |
| Number of full-term pregnancies | | | | | | | | |
| 0 | 23/15 | 1.00 | | 25/19 | 1.00 | | 1.00 | |
| 1–2 | 62/60 | 0.64 | (0.29–1.41) | 76/62 | 0.91 | (0.43–1.92) | 0.76 | (0.44–1.29) |
| ⩾3 | 69/79 | 0.55 | (0.24–1.25) | 72/92 | 0.58 | (0.28–1.19) | 0.55 | (0.32–0.93) |
| Trend | | $P=0.18$ | | | $P=0.046$ | | $P=0.010$ | |
| Number of incomplete pregnancies | | | | | | | | |
| 0 | 102/99 | 1.00 | | 121/98 | 1.00 | | 1.00 | |
| 1–2 | 44/43 | 0.99 | (0.54–1.79) | 45/63 | 0.53 | (0.32–0.88) | 0.71 | (0.49–1.03) |
| ⩾3 | 8/12 | 0.41 | (0.14–1.23) | 7/12 | 0.42 | (0.14–1.27) | 0.45 | (0.21–0.97) |
| Trend | | $P=0.24$ | | | $P=0.007$ | | $P=0.012$ | |
| Number of full-term pregnancies | | | | | | | | |
| 0 | 23/15 | 1.00 | | 25/19 | 1.00 | | 1.00 | |
| 1 | 20/21 | 0.61 | (0.24–1.56) | 29/20 | 0.95 | (0.39–2.28) | 0.78 | (0.42–1.46) |
| 2 | 42/39 | 0.71 | (0.30–1.67) | 47/42 | 0.90 | (0.40–2.05) | 0.76 | (0.43–1.35) |
| 3 | 28/40 | 0.52 | (0.21–1.28) | 34/54 | 0.48 | (0.22–1.07) | 0.46 | (0.26–0.82) |
| 4 | 24/25 | 0.55 | (0.22–1.36) | 15/25 | 0.45 | (0.18–1.13) | 0.53 | (0.28–1.00) |
| 5 | 7/8 | 0.56 | (0.14–2.19) | 12/6 | 1.24 | (0.36–4.27) | 0.79 | (0.33–1.91) |
| ⩾6 | 10/6 | 1.23 | (0.29–5.16) | 11/7 | 1.36 | (0.42–4.42) | 1.24 | (0.51–3.01) |
| Number of incomplete pregnancies | | | | | | | | |
| 0 | 102/99 | 1.00 | | 121/98 | 1.00 | | 1.00 | |
| 1 | 29/29 | 1.15 | (0.58–2.29) | 32/36 | 0.68 | (0.38–1.24) | 0.85 | (0.55–1.31) |
| 2 | 15/14 | 0.79 | (0.31–2.00) | 13/27 | 0.31 | (0.13–0.73) | 0.53 | (0.30–0.96) |
| 3 | 4/8 | 0.30 | (0.07–1.35) | 1/10 | 0.00 | (UNK)[b] | 0.13 | (0.04–0.46) |
| 4 | 1/2 | 0.15 | (0.01–2.81) | 2/2 | 0.66 | (0.06–7.83) | 0.56 | (0.11–3.00) |
| ⩾5 | 3/2 | 1.22 | (0.16–8.99) | 4/0 | ∞ | (UNK)[b] | 3.58 | (0.70–18.31) |

[a]Adjusted for family history, total fat, alcohol, calcium, weight 10 years ago and activity level. [b]Confidence limits unknown.

**Table IV** Adjusted[a] effects of number of pregnancies, both full-term and incomplete, on risk of colon cancer, by subsite

| | *Final pregnancy variables in model*[b] | | | |
|---|---|---|---|---|
| | *Full term* | *(Full term)*$^2$ | *Incomplete* | *(Incomplete)*$^2$ |
| All subjects | | | | |
| Beta[a] | −0.356 | 0.055 | −0.537 | 0.093 |
| (s.e.) | (0.129) | (0.019) | (0.168) | (0.039) |
| *P* | 0.006 | 0.003 | 0.001 | 0.016 |
| Caecum to splenic flexure | | | | |
| Beta[a] | −0.286 | 0.043 | −0.358 | 0.042 |
| (s.e.) | (0.182) | (0.024) | (0.195) | (0.027) |
| *P* | 0.12 | 0.07 | 0.07 | 0.12 |
| Descending & sigmoid colon | | | | |
| Beta[a] | −0.467 | 0.080 | −1.019 | 0.228 |
| (s.e.) | (0.206) | (0.033) | (0.299) | (0.086) |
| *P* | 0.02 | 0.02 | 0.0006 | 0.008 |

[a]Adjusted for family history, total fat, alcohol, calcium, weight 10 years ago and activity level. [b]Both linear and quadratic forms of the continuous variables for number of full-term pregnancies and number of incomplete pregancies, all forced into model simultaneously along with variables listed in footnote a above.

imal tumours (*P* value for trend = 0.12) while distal tumours are no longer associated with age at natural menopause (Table VI). We did not collect information on the ovarian status of hysterectomised women; we are, therefore, unable to comment on the effects of artificial menopause (bilateral ovariectomy). Hormone replacement therapy, however, is not associated with risk (Table V).

Since the effects of pregnancy, as described by the four-term model in Table IV, appear to be somewhat greater in the distal than in the proximal colon, we sought to determine if this difference could account for the crossover in male-to-female incidence rates in the distal colon. This was done by a three-step process. First, the expected overall pregnancy effects in the proximal and distal colon were estimated by using the respective coefficients (betas) from Table IV to model the pregnancy effects in each segment, using our neighbourhood controls to represent the general population (see Appendix for methods of computation). These overall expected pregnancy effects were 0.73 and 0.47 for the proximal and distal colon respectively. Second, expected incidence rates among never-pregnant females were estimated by 'removing' the overall protective effects of pregnancy from the female incidence rates in Table I (by dividing each age-specific rate by the appropriate subsite-specific pregnancy effect). Finally, age-specific ratios of males-to-never-pregnant females were computed to see if the crossover effect in the distal colon remained after the pregnancy effects were removed. Whereas the male-to-female ratios in Table I increased with age from roughly 0.8 to 1.6, the male-to-never-pregnant ratios remained virtually constant at roughly 0.8. In the proximal colon, where the effect of pregnancy was weaker, the ratios between men and never pregnant women also remained fairly constant (from 1.2 to 0.9).

### Discussion

This study provides evidence in support of a protective effect of pregnancy on colon cancer risk, and more specifically a trend of increasing protection with increasing number of pregnancies when the number of pregnancies is categorised as zero, one to two, and three or more. Most previous studies of pregnancy and colon cancer have focused on livebirths only, and these studies vary in quality as well as in outcome. Table VII summarises those studies we regard as adequate in design, i.e. involving population-based cases, community controls, non-trivial numbers and no apparent flaws in execution. Even though the two studies conducted in Canada failed to find any protection associated with pregnancy, and the results across studies are significantly heterogeneous, when our own data are combined with these presented in Table VII, a protective pattern remains–the overall RRs for one to two and three or more pregnancies respectively are 0.88 ($P=0.23$) and 0.76 ($P<0.001$).

Several of the studies described in Table VII were limited to women who had ever been married. In the present study, 56% and 58% of the never pregnant cases and controls respectively had ever been married, indicating that infertility *per se* does not appear to be a factor. Therefore, while studies limited to ever married women have reduced power, their outcomes should not be affected by restricting attention to married women.

In the present study, when the continuous rather than categorised variable for number of pregnancies was examined, the relationship between pregnancies and colon cancer risk was U-shaped. Whether this is generally true is not known, since investigators of previous positive studies have not reported their data after three pregnancies in sufficient detail. This needs to be investigated further.

We also observed independent U-shaped curves for both full-term pregnancies and pregnancies carried less than 7 months (including all known miscarriages and abortions, both spontaneous and induced). Published reports of only three previous studies mentioned pregnancies that did not result in a livebirth and in two of these the investigators suggested that non-livebirth outcomes may be a risk factor for colon cancer (Howe *et al.*, 1985; Potter & McMichael, 1983). Howe *et al.* (1985) reported a non-significant odds ratio (OR) of 1.8 for any versus no non-livebirth (excluding abortions and including stillbirths). Potter and McMichael (1983) reported that three cases and four controls had been pregnant but never produced a livebirth, giving a non-significant OR of 3.6 when the reference category was women whose first livebirth was before age 22; however, the crude OR is 1.0 when never pregnant women are used as the reference category. Finally, Weiss *et al.* (1981) found 'no case–control differences for pregnancies that were not full-term', but no numbers were cited.

We found weak, non-significant, U-shaped relationships with both age at first pregnancy and age at first livebirth. Of the previous studies described in Table VII, three reported increasing risk with age at first livebirth (Kune *et al.*, 1989; Potter & McMichael, 1983) or age at first pregnancy (Howe *et al.*, 1985); but three others (Miller *et al.*, 1980; Weiss *et al.*, 1981; Wu *et al.*, 1987) reported no effects.

In the present study, there was a weak trend for increasing protection from proximal colon cancer with increasing age at natural menopause (Table VII). A protective effect for a late menopause was observed in at least one prior study, which also found excess risk associated with hysterectomy (Wu *et al.*, 1987). The present study, however, supports two additional studies (Potter & McMichael, 1983; Weiss *et al.*, 1981) which found no excess risk linked to hysterectomy.

Our failure to observe an effect for age at menarche is consistent with the negative findings of a cohort study conducted in a retirement population (Wu *et al.*, 1987). Past use of oral contraceptives has not been extensive in the older women comprising this or previous study populations, so it is not surprising that the two previous studies reporting on this variable found non-significant but opposite effects (Potter & Michael, 1983; Weiss *et al.*, 1981), and we found non-significant but opposing effects for the two subsites. In contrast, other female hormones, most notably in the form of oestrogen replacement therapy, are widely used but have never been linked to risk of colon cancer, in either this or previous studies (Potter & McMichael, 1983; Weiss *et al.*,

**Table V** Adjusted[a] matched relative risks (and 95% confidence intervals) by subsite for menstrual history and use of oral contraceptives and other female hormones

| | Caecum to splenic flexure | | | Descending & sigmoid colon | | | All subsites | |
|---|---|---|---|---|---|---|---|---|
| | Matched case/control | RR[a] | (95% CI) | Matched case/control | RR[a] | (95% CI) | Matched RR[a] | (95% CI) |
| Age at menarche | | | | | | | | |
| <12 | 30/26 | 1.00 | | 39/37 | 1.00 | | 1.00 | |
| 12 | 36/40 | 0.75 | (0.33–1.70) | 32/36 | 0.94 | (0.42–2.08) | 0.81 | (0.47–1.39) |
| 13 | 49/38 | 1.06 | (0.47–2.39) | 53/45 | 1.10 | (0.58–2.08) | 1.13 | (0.70–1.82) |
| >13 | 39/50 | 0.65 | (0.29–1.48) | 49/55 | 0.88 | (0.44–1.78) | 0.77 | (0.46–1.28) |
| Years of using oral contraceptives | | | | | | | | |
| Never | 123/127 | 1.00 | | 145/138 | 1.00 | | 1.00 | |
| <5 | 24/19 | 1.42 | (0.62–3.30) | 22/28 | 0.70 | (0.32–1.53) | 1.02 | (0.59–1.75) |
| ⩾5 | 7/8 | 1.30 | (0.31–5.46) | 6/7 | 0.98 | (0.20–4.82) | 1.06 | (0.39–2.89) |
| Age and type of menopause | | | | | | | | |
| Age at natural menopause | | | | | | | | |
| ⩽47 | 28/14 | 2.27 | (0.93–5.53) | 20/37 | 0.40 | (0.16–0.93) | 0.94 | (0.54–1.65) |
| 48–52 | 37/44 | 1.00 | | 46/31 | 1.00 | | 1.00 | |
| ⩾53 | 31/40 | 0.60 | (0.28–1.27) | 31/34 | 0.70 | (0.34–1.45) | 0.71 | (0.43–1.16) |
| Age at hysterectomy | | | | | | | | |
| ⩽47 | 42/41 | 1.17 | (0.56–2.46) | 54/48 | 0.85 | (0.44–1.65) | 1.02 | (0.64–1.63) |
| 48–52 | 10/6 | 1.51 | (0.40–5.74) | 14/13 | 0.71 | (0.25–1.98) | 1.06 | (0.49–2.26) |
| Premenopausal | | | | | | | | |
| | 6/9 | 0.61 | (0.13–2.81) | 8/10 | 0.62 | (0.15–2.59) | 0.64 | (0.24–1.71) |
| Years of hormone replacement therapy[b] | | | | | | | | |
| Never | 66/72 | 1.00 | | 76/82 | 1.00 | | 1.00 | |
| <5 | 56/44 | 1.44 | (0.80–2.62) | 50.43 | 1.25 | (0.69–2.28) | 1.32 | (0.88–1.98) |
| 5–14 | 16/20 | 1.09 | (0.47–2.56) | 30/30 | 1.10 | (0.55–2.21) | 1.08 | (0.64–1.82) |
| ⩾15 | 16/18 | 1.19 | (0.51–2.78) | 17/18 | 0.75 | (0.30–1.85) | 1.05 | (0.58–1.89) |

[a]Adjusted for family history, total fat, alcohol, calcium, weight 10 years ago, activity level and pregnancy (linear and quadratic terms of both full-term and incomplete pregnancies). [b]Adjusted for type and age of menopause as well as factors listed above.

**Table VI** Adjusted[a] relative risks (and 95% confidence intervals) by subsite for age and type of menopause, using all controls[b] at each subsite

| | Caecum to splenic flexure | | | Descending & sigmoid colon | | |
|---|---|---|---|---|---|---|
| | Case/Control | RR[a] | (95% CI) | Case/control | RR[a] | (95% CI) |
| Age at natural menopause | | | | | | |
| ⩽47 | 28/51 | 1.22 | (0.64–2.35) | 20/51 | 0.66 | (0.34–1.29) |
| 48–52 | 37/75 | 1.00 | | 46/75 | 1.00 | |
| ⩾53 | 31/74 | 0.68 | (0.36–1.28) | 31/74 | 0.63 | (0.35–1.14) |
| Age at hysterectomy | | | | | | |
| ⩽47 | | | | | | |
| 48–52 | 42/89 | 0.86 | (0.48–1.55) | 54/88 | 0.99 | (0.58–1.68) |
| | 10/19 | 1.20 | (0.49–2.98) | 14/19 | 1.37 | (0.61–3.12) |
| Premenopausal | | | | | | |
| | 6/19 | 0.41 | (0.12–1.37) | 8/19 | 0.59 | (0.20–1.72) |

[a]Adjusted for family history, total fat, alcohol, calcium, weight 10 years ago, activity level and pregnancy (linear and quadratic terms of both full-term and incomplete pregnancies). [b]Compared to cases at each subsite in a stratified analysis, matching within age-social class strata: ages ⩽54, 55–64 and ⩾65 and three socio-economic strata.

**Table VII** Summary of 'adequate'[a] studies of parity (or pregnancies) and colon cancer

| *Citation* | *Location* | *Live-births*[b] | *Cases/controls*[c] | *RR*[2] | *Comments* |
|---|---|---|---|---|---|
| Miller *et al.* (1980) | Canada | 0 | 71/N[d] | 1.0 | Population-based cases compared to census data. Limited to ever married women. Collected parity data by mail. RR's adjusted for age. Found expected parity effect for ovarian, endometrial and *in situ* cervix cancer but not for breast or invasive cervix cancers after adjusting for age 1st pregnancy. |
| | | 1–2 | 217/2.6N[d] | 1.16 | |
| | | ⩾3 | 465/5.5N[d] | 1.20 | |
| Weiss *et al.* (1981) | Western Washington State | 0 | 22/107 | 1.0 | Population-based cases with community controls. Limited to white women ages 46–74. RRs adjusted for age. |
| | | 1–2 | 48/286 | 0.7 (0.4–1.3) | |
| | | ⩾3 | 25/313 | 0.5 (0.3–0.8) Trend = 0.004 | |
| Potter *et al.* (1983) | South Australia | 0 | 17/33 | 1.0 | Population-based cases with matched community controls. Limited to women ages 30–74. Unmatched ORs shown. |
| | | 1–2 | 50/129 | 0.9 (0.4–1.8) | |
| | | ⩾3 | 32/149 | 0.4 (0.2–0.8) | |
| McMichael & Potter (1984) | South Australia | 0 | 79/15,500 | 1.0 | Population-based deaths with all living women as controls. Limited to ever married women. RRs adjusted for age. Same pattern but decreased magnitude of effect seen when colon cancer deaths compared to all other deaths. |
| | | 1–2 | 230/82,720 | 0.72 (0.56–0.93) | |
| | | ⩾3 | 206/105,498 | 0.63 (0.48–0.81 | |
| Howe *et al.* (1985) | Canada | 0 | 18/19 | 1.0 | 'Population'-based cases compared to matched neighbourhood controls. Limited to ever married women. |
| | | 1–2 | 66/64 | 1.10 | |
| | | ⩾3 | 74/85 | 0.97 | |
| Wu *et al.* (1987) | Los Angeles California | 0 | 20/2,422 | 1.0 | 4-year follow-up of 11,888 residents of retirement community. RRs adjusted for age. |
| | | 1–2 | 33/3,767 | 1.06 | |
| | | ⩾3 | 5/1,205 | 0.54 n.s. | |
| Kune *et al.* (1989) | Melbourne, Australia | 0 | 60/45 | 1.0 | Population-based cases compared to community controls frequency matched on age. Includes rectal cancer. |
| | | 1–2 | 116/124 | 0.70 Trend = | |
| | | ⩾3 | 129/158 | 0.61 0.04 | |
| Overall (including present data) | All of above | 0 | 328/- | 1.0 | Meta-analysis of data from above 7 studies plus present study, using Mantel–Haenszel method of combining data (Breslow & Day, 1980). |
| | | 1–2 | 877/- | 0.88 (0.76,1.02) | |
| | | ⩾3 | 1,105/- | 0.76 (0.66,0.88) Trend = 0.0001 | |

[a]Population-based cases, community controls, non-trivial numbers, and no obvious errors in execution. [b]All studies based on livebirths except for Howe *et al.* (1984) and the present one, which were based on all pregnancies. [c]Some of the numbers are approximate due to weighting of figures given in the specified papers. [d]N = large number; based on census data.

1981; Wu *et al.*, 1987).

The relationships observed in this study between number of pregnancies and risk of colon cancer are not easily explained by bias or confounding. Recall bias is not likely since both case and matching control were asked about their pregnancies in the same structured manner by the same interviewer, and neither subjects nor interviewers were aware of any hypotheses linking reproductive history to cancer risk. Selection bias is not likely since our response rates in both cases and controls were high, women reporting their occupations as 'housewives' were equally represented among cases and controls, and we can think of no reason for cases with three to five pregnancies (but neither fewer nor more) to be under-represented in the case series and/or over-represented in the control series, and for this to be true for incomplete as well as for full-term pregnancies. To avoid confounding, we adjusted for all factors associated with risk in this study, as well as for a number of variables not associated with risk, such as smoking, alcohol intake, education, income, age at first pregnancy and age at first livebirth.

The male-to-female ratios of the age-specific incidence rates observed in Los Angeles County (Table I) are consistent with those previously reported for other Western countries (McMichael & Potter, 1980). In addition, our observation that the crossover in these rates is limited to the descending and sigmoid colon has also been reported previously for combined data from seven Caucasian populations (McMichael & Potter, 1983).

The crossover of male and female incidence rates in the distal colon could be due to a protective effect of pregnancy. This hypothesis is supported by the ability of our modelled pregnancy effect to explain completely the crossover phenomenon. While we did observe a pregnancy effect in the proximal colon, it was weaker and less consistent at that site.

Several mechanisms have been suggested to explain a protective effect of pregnancy on colon cancer, including hormonal influences on bile metabolism (McMichael & Potter, 1980), immunological influences of ABO-incompatible fetal antigens (Bjelke, 1973, 1974), increased physical activity associated with large families (Wu *et al.*, 1987) and 'as yet unidentified' lifestyle factors associated with having children (Kune *et al.*, 1989). The latter two hypotheses were proposed when the 'parity' effect was observed in men as well as in women (Kune *et al.*, 1989; Wu *et al.*, 1987). We have no family size information on men, but controlling for activity level did not alter the pregnancy effects in our female subjects. This was true whether physical activity was based on 5 or 30 years before diagnosis. We also found an independent effect for incomplete pregnancies, which should have no effect on activity levels. The ABO-incompatible fetal antigen hypothesis was suggested when the protective effect of multiple pregnancies in two parallel case–control studies was limited to women with blood group O (Bjelke, 1973, 1974). We have no data on blood group but there is little evidence that immune factors play a role in large bowel cancer (Hill, 1981). The bile acid mechanism was suggested by McMichael and Potter (1980). Published data on the effects of pregnancy on human bile composition in the duodenum are not consistent (Bennion & Grundy, 1978; Nakagaki, & Nakayama, 1982), and there are no published data on the effects of pregnancy on the level or composition of bile acids in the colon itself. However, both progesterone and pregnancy do appear to decrease gallbladder emptying (Bennion & Grundy, 1978; Nakagaki & Nakayam, 1982), which may reduce the level of bile acids in the colon.

It is also possible, however, that the hormones of pregnancy have a direct effect on colonic mucosa which ultimately leads to lower risk of colon cancer. Oestrogen receptors occur in measurable quantities in both human colon carcinoma and in surrounding non-cancerous colonic tissue (Francavilla *et al.*, 1987). Significant levels of progesterone receptors have also been measued in malignant colorectal tumors (Sica *et al.*, 1984), but the actual effects of these hormones on human colonic cells is not known. In mice, the populations of both proliferative and differentiated columnar cells lining the colonic crypts vary as a function of the oestrogen cycle (Hoff & Chang, 1979), and experiments suggest that progesterone, which peaks after ovulation, promotes differentiation of epithelial cells in the colonic crypt and may also serve to maintain these differentiated cells while simultaneously inhibiting proliferation (Hoff & Chang, 1979). If so, the high levels of progesterone occurring during pregnancy, even pregnancies interrupted by miscarriage or abortion, may reduce proliferation while maintaining a relatively larger population of differentiated epithelial cells. Since differentiated cells are generally less susceptible to initiation and promotion of carcinogenesis than dividing cells (Chang, 1981), the mother may subsequently be protected.

If indeed progesterone is the basis for the protective effect of pregnancy, one might expect to find a protective effect of OC use. The progestational effect of OC use, however, is considerably smaller than that of pregnancy; and the absence of a protective OC effect may indicate that very high progesterone levels are needed to produce a noticeable degree of protection.

To our knowledge, only one animal study has examined colon cancer incidence in relation to prior pregnancies (Sjogren, 1977). Here multiparous rats formed significantly fewer 1,2-dimethylhydrazine (DMH)-induced tumours than age-matched virgin female rats. Although this finding lends credence to a protective effect of pregnancy itself, as opposed to some lifestyle factor associated with raising children, the author attributes the protective effect not to hormonal influences but to the immunity of multiparous females to embryonal antigens present on colorectal carcinomas, since rats previously inoculated with isografts of DMH-induced colon carcinomas showed the same low rates of tumour incidence as the multiparous females, while rats inoculated with isografts of mammary tumours or *N*-methyl-*N'*-nitrosoguanidine-induced colon tumours had tumour rates comparable to the untreated controls.

If the U-shape of the pregnancy effect is not an artefact of this study, then it suggests competing factors, some protective and others conferring increasing risk with successive pregnancies. For example, multiple full-term pregnancies may negate the beneficial effects of the first three or four pregnancies through cumulative non-specific injuries caused by compression of the large bowel by a growing fetus, particularly in the distal portion, and possible traumatic injuries to the rectosigmoid during delivery. Non-specific injuries are known to promote colonic carcinogenesis in rats (Pozharisski, 1975). Multiple incomplete pregnancies may negate the beneficial effects of pregnancy for different reasons, perhaps related to the constipating effects of pregnancy, which are presumably created by the high levels of progesterone released almost from the onset of the pregnancy, or to unknown biological characteristics of women who have difficulty carrying a fetus to full-term.

Even though the age-specific incidence rates for colon cancer in men and women are highly correlated and roughly equal in most populations, the crossover of these rates in the distal colon and the effects of parity observed here and elsewhere are intriguing. Clearly there is something about being female which protects women from distal colon cancer after age 55, and something about pregnancy, whether completed or not, which influences a woman's risk of colon cancer. Opposing competing factors could not only produce the U-shaped pregnancy effect observed here but may explain the lack of consistency in the parity effect across studies conducted in different populations. The existence of such competing factors, be they bile acid concentration, direct effects of progesterone, immunological processes or other factors, needs to be investigated. Other studies with large numbers of women are needed to confirm the U-shaped curve observed here; at the same time, studies of the effects of both normal pregnancy and large doses of progesterone on proliferation rates of colonic cells could be most helpful in clarifying the role of pregnancy in colon cancer risk.

### Appendix

### *Method of estimating overall pregnancy effects*

To estimate the overall pregnancy effects in the proximal and distal colon, two assumptions were made. First, these pregnancy effects were assumed to be independent of age since no significant interaction with age was found with the pregnancy parameter in this dataset; and second, the frequency distribution of full-term and incomplete pregnancies among controls was assumed to represent the proportion of women in the general population in each cell of the full-term/incomplete pregnancy matrix. For each cell in this pregnancy matrix (e.g. two full-term pregnancies and one incomplete pregnancy), we calculated the expected cell-specific pregnancy effect, i.e. the expected colon cancer rate relative to the rate in a population of women who were never pregnant, using the subsite-specific four-term model from Table IV. These rate ratios were then multiplied by the proportion of the control population represented by the respective cell, and the products were summed to estimate the overall expected pregnancy effect in the respective segment of the colon.

We are indebted to Dawn Elliot, Mary Davis, Monica Garfield and Susan Roberts for assistance in collecting and coding the data for this study; and to Jerzy Lysikowski for assistance with data analysis. Supported by grants from the National Cancer Institute (CA36501, CA44401, CA17054 and CA14089). Cancer incidence data were collected under contract to the California Department of Health Services as mandated by the Health & Safety Code Section 211.3.

### References

ACHESON, R.M., CHAPPEL, M. & EISENBERG, H. (1975). Infertility as a risk factor for carcinoma of the colon in women. Cited by B.S. Schoenberg, Multiple primary neoplasms. In *Persons at High Risk of Cancer: an Approach to Etiology and Control*, Fraumeni, J.F. (ed.) p. 111. Academic Press: New York.

ASTRIN, S.M. & COSTANZI, C. (1989). The molecular genetics of colon cancer. *Semin. Oncol.*, **16**, 138.

BENNION, L.J. & GRUNDY, W.M. (1978). Risk factors for the development of cholelithiasis in man. *N. Engl. J. Med.*, **229**, 1221.

BJELKE, E. (1973). Colorectal cancer: clues from epidemiology. International Cancer Congress Series No. 354. *Excerpta Med.*, **6**, 324.

BJELKE, E. (1974). Epidemiologic studies of cancer of the stomach, colon, and rectum. *Scand. J. Gastroenterol.*, **9**, suppl. 31, 1.

BRESLOW, N.E. & DAY, N.E. (1980). *Statistical Methods in Cancer Research*. International Agency for Research on Cancer: Lyon.

BYERS, T., GRAHAM, S. & SWANSON, M. (1982). Parity and colorectal cancer risk in women. *J. Natl Cancer Inst.*, **69**, 1059.

CHANG, W.W.L. (1981). Degenerative behavior of epithelial cells in the colonic crypt of the mouse following administration of colonic carcinogen, 1,2-dimethyhydrazine. *Cancer Lett.*, **13**, 111.

DALES, L.G., FRIEDMAN, G.D., URY, H.K., GROSSMAN, S. & WILLIAMS, S.R. (1978). A case-control study of relationships of diet and other traits to colorectal cancer in American Blacks. *Am. J. Epidemiol.*, **109**, 132.

FRANCAVILLA, A., DILEO, A., POLIMENO, L. & 6 others (1987). Nuclear and cytosolic estrogen receptors in human colon carcinoma and in surrounding noncancerous colonic tissue. *Gastroenterology*, **93**, 1301.

HILL, M.J. (ed.) (1981). *Metabolic Epidemiology of Large Bowel Cancer*. Martinus Nijhoff: Boston.

HOFF, M.B. & CHANG, W.W.L. (1979). The effect of estrogen on epithelial cell proliferation and differentiation in the crypts of the descending colon of the mouse: a radioautographic study. *Am. J. Anat.*, **155**, 507.

HOWE, G.R., CRAIB, K.J.P. & MILLER, A.B. (1985). Age at first pregnancy and risk of colorectal cancer: a case-control study. *J. Natl Cancer Inst.*, **74**, 1155.

KUNE, G.A., KUNE, S. & WATSON, L.F. (1989). Children, age at first birth, and colorectal cancer risk. *Am. J. Epidemiol.*, **129**, 533.

LAMBERT, R. (1982). Epidemiology of colorectal carcinogenesis. In *Colonic Carcinogenesis*, Malt, R.A. & Williamson, R.C.N. (eds) p. 10. MTP Press: Hingham, MA.

MACK, T.M. (1977). Cancer surveillance program in Los Angeles County. *Natl Cancer Inst. Monogr.*, **47**, 99.

MCMICHAEL, A.J. & POTTER, J.D. (1980). Reproduction, endogenous and exogenous sex hormones, and colon cancer: a review and hypothesis. *J. Natl Cancer Inst.*, **65**, 1201.

MCMICHAEL, A.J. & POTTER, J.D. (1983). Do intrinsic sex differences in lower alimentary tract physiology influence the sex-specific risks of bowel cancer and other biliary and intestinal diseases? *Am. J. Epidemiol.*, **118**, 620.

MCMICHAEL, A.J. & POTTER, J.D. (1984). Parity and death from colon cancer in women: a case-control study. *Comm. Health Studies*, **8**, 19.

MILLER, A.B., BARCLAY, T.H.C., CHOI, N.W. & 6 others (1980). A study of cancer, parity and age at first pregnancy. *J. Chron. Dis.*, **33**, 595.

NAKAGAKI, M. & NAKAYAMA, F. (1982). Effect of female sex hormones on lithogenicity of bile. *Jap. J. Surg.*, **12**, 13.

PAPADIMITROU, C., DAY, N., TZONOU, A., GEROVASSILIS, F., MANOUSOS, O. & TRICHOPOULOS, D. (1984). Biosocial correlates of colorectal cancer in Greece. *Int. J. Epidemiol.*, **13**, 155.

PETERS, R.K., GARABRANT, D.H., PIKE, M.C. & MACK, T.M. (1990*a*). Physical activity body size, and family history in the etiology of colon cancer (in the press).

PETERS, R.K., GARABRANT, D., YR, M. & MACK, T.M. (1989). A case-control study of occupational and dietary factors in colorectal cancer in young men by subsite. *Cancer Res.*, **149**, 5459.

PETERS, R.K., PIKE, M.C., GARABANT, D.H. & MACK, T.M. (1990*b*). Fat, calcium, and other dietary factors in the etiology of colon cancer (in preparation).

PLESKO, I., PRESTON-MARTIN, S., DAY, N.E., TZONOU, A., DIMITROVA, E. & SOMOGYI, J. (1985). Parity and cancer risk in Slovakia. *Int. J. Cancer*, **36**, 529.

POTTER, J.D. & MCMICHAEL, A.J. (1983). Large bowel cancer in women in relation to reproductive and hormonal factors: a case-control study. *J. Natl Cancer Inst.*, **71**, 703.

POZHARISSKI, K.M. (1975). The significance of nonspecific injury for colon carcinogenesis in rats. *Cancer Res.*, **35**, 3824.

SICA, V., NOLA, E., CONTIERI, E. & 6 others (1984). Estradiol and progesterone receptors in malignant gastrointestinal tumors. *Cancer Res.*, **44**, 4670.

SIEGAL, J.S. (1973). *Estimates of Coverage of the Population by Sex, Race, and Age in the 1970 Census*. Proceedings of the Annual Meeting, Population Association of America: Washington, DC.

SJOGREN, H.O. (1977). Overview: the application of immunology to the development of immunotherapeutic programs for patients with large bowel cancer. *Cancer*, **40**, 2710.

WEISS, N.S., DALING, J.R. & CHOW, W.H. (1981). Incidence of cancer of the large bowel in women in relation to reproductive and hormonal factors. *J. Natl Cancer Inst.*, **67**, 57.

WU, A.H., PAGANINI-HILL, A., ROSS, R.K. & HENDERSON, B.E. (1987). Alcohol, physical activity, and other risk factors for colorectal cancer: a prospective study. *Br. J. Cancer*, **55**, 687.